# Peri-Operative Hypothermia in Trauma Patients: A Retrospective Cohort Analysis at a Busy District General Hospital Within the National Health Service (NHS)

**DOI:** 10.7759/cureus.74979

**Published:** 2024-12-02

**Authors:** Zain Habib, Mohammed Arifuzaman, Ahmed Elbeltagi, Apurv Gupta, Shua Haq, Dhiman Sikder, Muhammad Umer Rasool, Swapnil M Saraiya, Syed Ali Abbas Bilgrami, Muhammed Suneer Puthan Peedika, Sayan Bhattacharya, Mahdi Khalfaoui, Parth B Gada

**Affiliations:** 1 Trauma and Orthopaedics, Manchester University National Health Service (NHS) Foundation Trust, Manchester, GBR; 2 General Surgery, North Manchester General Hospital, Manchester, GBR; 3 General and Colorectal Surgery, Manchester University National Health Service (NHS) Foundation Trust, Manchester, GBR; 4 Trauma and Orthopaedics, Wythenshawe Hospital, Manchester, GBR; 5 Orthopaedics, North Manchester General Hospital, Manchester, GBR; 6 General Surgery, University Hospital of North Tees, North Tees and Hartlepool National Health Service (NHS) Foundation Trust, Stockton-on-Tees, GBR; 7 Internal Medicine, North Manchester General Hospital, Manchester, GBR; 8 Emergency Medicine, Manchester University National Health Service (NHS) Foundation Trust, Manchester, GBR; 9 Trauma and Orthopaedics, North Manchester General Hospital, Manchester, GBR

**Keywords:** intra-operative hypothermia, operating theatre temperature, peri-operative hypothermia, pre-warming, trauma and orthopaedic surgery

## Abstract

Introduction: Perioperative hypothermia is defined as a patient's core body temperature of less than 36°C, which can lead to several complications. Even mild hypothermia increases the incidence of post-operative wound infection, post-operative ischaemic cardiac events and intra-operative blood loss and prolongs post-operative recovery. It is, hence, essential to maintain and provide normothermia during the perioperative phases for optimal surgical results and patient satisfaction. One of the most significant contributing factors to intra-operative hypothermia is the induction of general anaesthesia, where a significant amount of heat is shifted from the core to the peripheral circulation with consequent loss to an often-cold environment. The difference between the patient's skin and ambient temperature during the interval from entering the operating room through anaesthesia induction until draping and active warming may be significant. This study aims to look at the incidence of perioperative hypothermia in trauma and orthopaedics patients who present to a busy district general hospital in the National Health Service (NHS) and correlate this with the ambient theatre temperature and phases of surgery to draw a statistical significance.

Methods: This retrospective observational study conducted at the North Manchester General Hospital's trauma and orthopaedics department included 300 patients listed in the trauma surgery list from 1 July 2023 to 31 August 2023. Inclusion criteria were trauma patients aged 16-85 years. Elective orthopaedic and other surgical speciality patients were excluded. The perioperative temperature measurements were collected from the anaesthesia records. Statistical calculations were conducted using the StatsDirect software (StatsDirect Ltd, Wirral, UK) from Manchester University NHS Foundation Trust, Manchester.

Results: Among the 300 patients, the overall incidence of hypothermia was 3% pre-operative, 18% pre-induction, 21% intra-operative, 21% post-operative, 3% in recovery and 0% post-recovery. Intra-operative hypothermia incidence was significant, given that active warming was applied to patients with pre-operative hypothermia. Multivariate regression analysis showed that pre-induction temperature and theatre ambient temperature were statistically significant in predicting intra-operative hypothermia.

Conclusion: This study highlights the need for active interventions to recognise and prevent perioperative hypothermia in trauma and orthopaedics patients. Active pre-warming of patients and the operating rooms, regardless of surgery type and duration, is feasible and potentially beneficial. Further studies should include a randomised controlled trial comparing active and passive warming strategies to evaluate their effectiveness in improving perioperative outcomes.

## Introduction

The importance of maintaining normothermia, defined as a core body temperature between 36.5°C and 37.5°C, during the perioperative phase is well established. Perioperative hypothermia is defined as a patient's core body temperature of less than 36°C. It can lead to several complications, and even mild hypothermia increases the incidence of post-operative wound infection [1,2], post-operative ischaemic cardiac events [3] and intra-operative blood loss [4,5], and prolongs post-operative recovery [6,7]. It is, hence, essential to maintain and provide normothermia during the perioperative phases for optimal surgical results and patient satisfaction. The incidence of perioperative hypothermia varies widely and can range from 4% to greater than 70% [8,9]. There is strong evidence to suggest that a direct relationship exists between a decreasing core body temperature and increased mortality rates in trauma patients, and hypothermia, acidosis and coagulopathy form a "Lethal Triad" [10-17].

Perioperative hypothermia is a common occurrence due to core-to-peripheral heat redistribution, aesthetic-related impaired thermoregulation, use of irrigation fluids, loss of barrier protection of skin and exposure of tissues to the environment [18-21]. Perioperative heat loss occurs through radiation, conduction, convection and evaporation, and radiation accounts for 60% of total heat loss [20,22]. The most common mechanism of accidental hypothermia is convective heat loss to cold air and conductive heat loss to water [20]. One of the most significant contributing factors to intra-operative hypothermia is the induction of general anaesthesia, where a significant amount of heat is shifted from the core to the peripheral circulation with consequent loss to an often-cold environment. The difference between the patient's skin and ambient temperature during the interval from entering the operating room through anaesthesia induction until draping and active warming may be significant. Without any active intervention, the patient's impaired defences against hypothermia persist. In an unwarmed surgical patient, the core body temperature continues to reduce because of a negative balance of heat loss to metabolic heat production until an equilibrium is reached at around 33°C core body temperature [23].

Operative hypothermia is classed as mild if the core body temperature is below 36°C, moderate if below 34°C and severe if below 32°C. Jurkovich et al. [24] reported a 100% mortality rate in trauma patients whose admission temperatures were below 32°C.

Many products have been used pre-operatively and intra-operatively to prevent hypothermia but are normally not activated until the patient has been anaesthetised, prepped and draped for surgical incision [25]. It is during this phase that the patient is at greatest risk of hypothermia [26]. The operating rooms are usually maintained at low temperatures, as warm rooms can be extremely uncomfortable for gowned surgeons and staff. Many studies have evaluated the efficacy of active warming devices in pre-warming patients before entering the operating room [2,25,27-29]. Sixty minutes of forced air warming device pre-warming has been shown to reduce the redistributive hypothermia on induction and reduce the incidence of perioperative hypothermia [26]. A study in paediatric patients showed that pre-warming induction and operating rooms led to a slower decline in the core body temperature of the patients [30]. This effect was diminished during longer cases with intra-operative warming. Pre-warming of the operating rooms in adult cases, similarly, may minimise the heat loss occurring after induction of general anaesthesia and before active patient warming [31].

The authors intend to study the incidence of perioperative hypothermia in various surgical phases of general trauma and orthopaedic patients in a busy NHS district general hospital, correlate this with the ambient theatre temperature and draw any statistical significance.

## Materials and methods

The study was registered with the North Manchester General Hospital (NMGH) audit and quality improvement department with registration number 10486. NMGH is a large district hospital in the north of Manchester that caters to a large population base. It runs a trauma list daily and caters to general trauma, including upper and lower limb trauma, excluding the pelvis.

Study design

This retrospective study included 300 patients listed in the trauma list at NMGH between 1 July 2023 and 31 August 2023. Inclusion criteria were trauma patients aged 16-85 years. Elective orthopaedic and other surgical speciality patients were excluded. Data collected included age, body mass index (BMI), American Society of Anaesthesiologists (ASA) score, surgery type, surgical time, anaesthesia time and median theatre ambient temperature. The temperature values were collected from the anaesthetic records of the patients, and the values used for the study included pre-operative temperature, pre-induction temperature, intra-operative, post-procedure, temperature in recovery and temperature post-recovery. The ambient operating room temperature was collected from the operating room records.

Pre-operative temperature was measured 1 hour before anaesthesia [32]. The pre-induction temperature is the core body temperature of the patient immediately before anaesthesia induction agents are administered. The intra-operative temperature was recorded as the median value of the continuous intra-operative temperature measurement. The post-procedure temperature value was the median temperature reading after the completion of the procedure and reversal of anaesthesia. The median core body temperature of the patient in the recovery room was recorded as the recovery temperature, and the core body temperature after shifting to the ward was the post-recovery temperature.

The temperature measurements were done via the tympanic thermometers during all the perioperative phases. All the patients were managed according to the Royal College of Anaesthesiologists' Guidelines for the Provision of Anaesthetic Services, and inadvertent perioperative hypothermia was managed according to the National Institute of Clinical Excellence guidelines 65 [32]. All the patients listed on the trauma list had their temperature checked before entering the anaesthesia suite, and all patients had blankets to cover them. Patients with a core body temperature of less than 36°C were offered active pre-warming with the use of a forced-air patient warming system (Bair Hugger, 3M Medical, St. Paul, MN), and the patients were anaesthetised only if core temperatures were greater than 36°C. Warm crystalloids were used for all patients.

Statistical calculations were performed using the StatsDirect software (StatsDirect Ltd, Wirral, UK) from Manchester University National Health Service (NHS) Foundation Trust. Multivariate regression analysis was conducted, with p <0.05 considered significant.

## Results

The data were collected in Excel sheets (Microsoft Corporation, Redmond, WA), and statistical calculations were performed on the StatsDirect software. Out of the 300 patients, 182 (60.6%) had upper limb trauma, and 118 (39.4%) had lower limb trauma. Out of the total patients, 177 (59%) were male patients and 123 (41%) were female patients. The median age of all the patients was 59.7 years.

**Figure 1:**
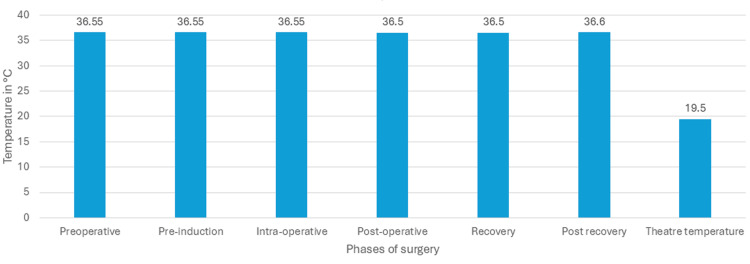
Median temperatures in various phases of surgery

The median pre-operative temperature was 36.55°C (mean, 36.57 °C; standard deviation, SD, 0.41), and intra-operative was 36.5°C (mean, 36.47 °C; SD, 0.39). Median ambient operating room temperature was recorded as 19.5 °C (mean, 19.4°C; SD, 0.38) (Figure 1).

**Figure 2:**
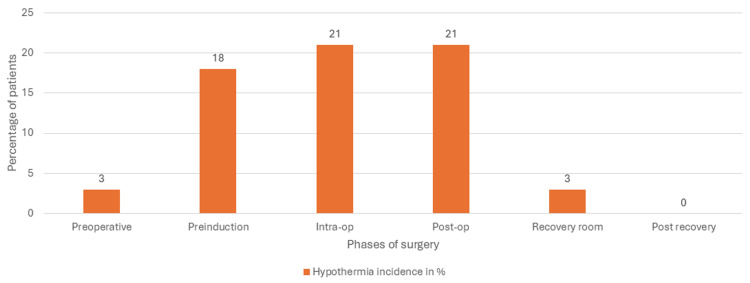
Percentage of incidence of hypothermia in trauma patients in various phases of surgery

We calculated the overall incidence of hypothermia for each phase of the surgery. Pre-operative hypothermia occurred in nine patients (3%), pre-induction hypothermia in 54 patients (18%), intra-operative hypothermia in 62 patients (21%), post-operative hypothermia in 62 patients (21%), recovery room hypothermia in nine patients (3%), and none of the patients developed post-recovery hypothermia (Figure 2). Of the 62 patients who developed intra-operative hypothermia, 32 (51.6%) were male and 30 (48.4%) were female patients.

**Figure 3:**
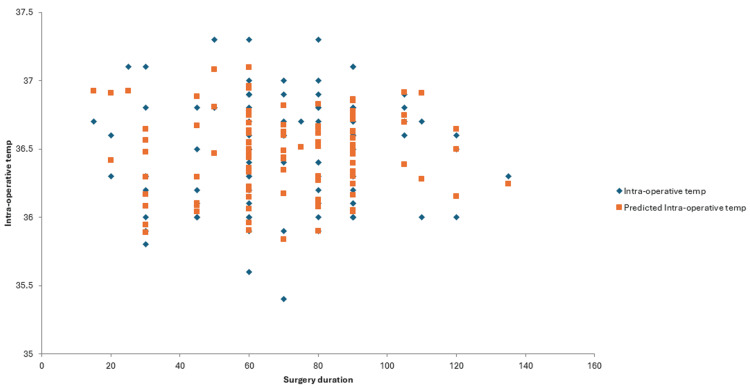
Line fit plot between duration of surgery (in minutes) and intra-operative patient temperatures (in °C)

Multivariate regression analysis of intra-operative temperature measurements and the duration of surgery resulted in a p value of 0.96, indicating no correlation between the two in our study. Figure 3 shows that the patients developed hypothermia in short as well as long surgical cases. Multivariate regression analysis of intra-operative temperature measurements, BMI, ASA categories, age and sex resulted in p values of 0.84, 0.40, 0.32 and 0.77, respectively, indicating no statistically significant relationship (Table 1).

**Table 1:** Patient variables and calculations The mean and SD have been rounded up to two decimal places p values are a result of multivariate regression analysis SD: standard deviation; BMI: body mass index; ASA: American Society of Anaesthesiologists

Variables	Mean	SD	Minimum	Maximum	p value
Intra-operative temperature (°C)	36.57	0.42	35.6	27.7	0.84
BMI	29.19	1.93	15.3	44.8	0.4
ASA grade	7.01	0.73	1	4	0.32

**Figure 4:**
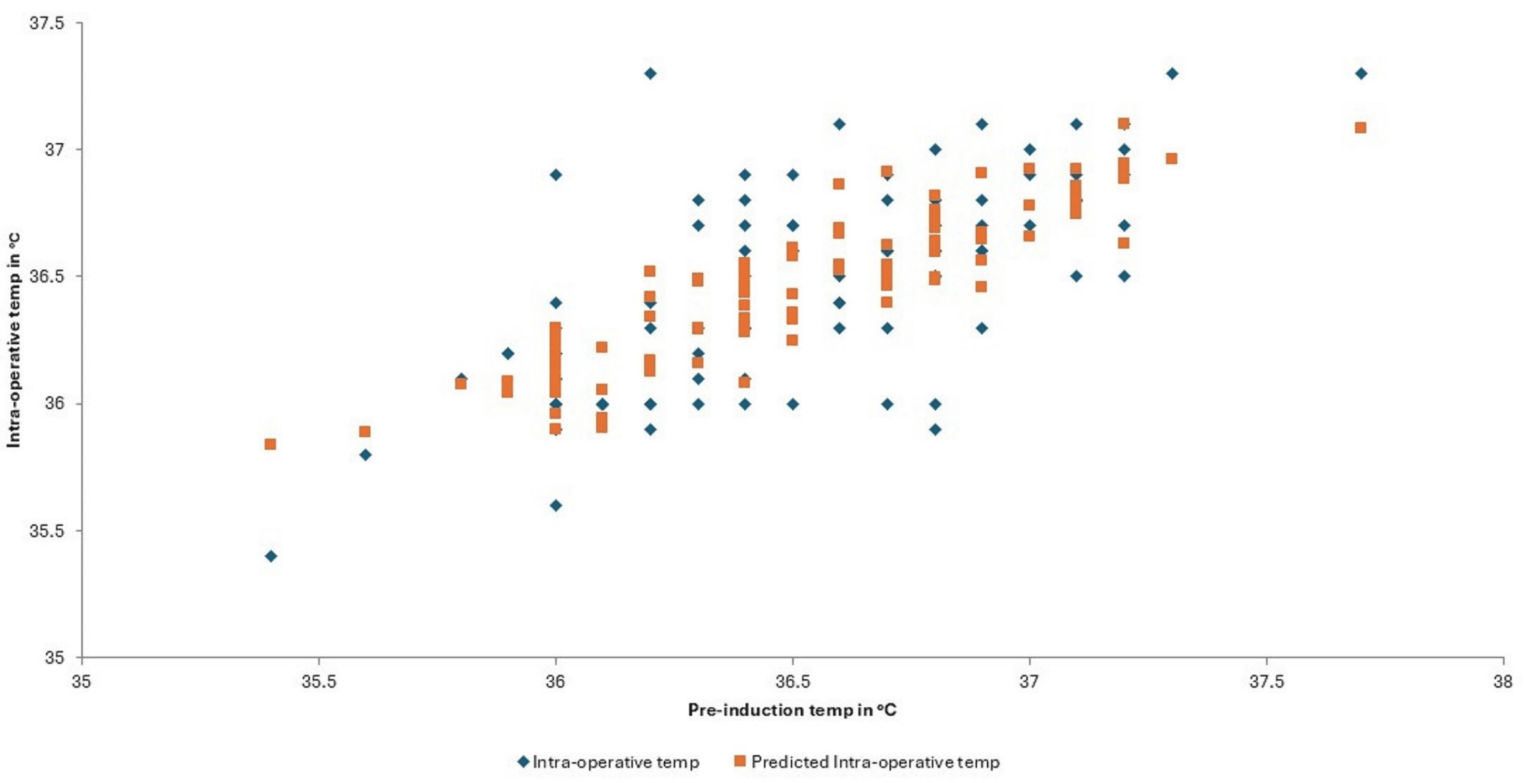
Line fit plot between pre-induction temperature and intra-operative temperature in trauma patients

Multivariate regression analysis of pre-operative, pre-induction and intra-operative temperatures resulted in a p value of 0.363 for pre-operative and <0.001 for pre-induction temperatures (Figure 4). This suggests that pre-induction temperatures are a statistically significant parameter for predicting intra-operative hypothermia. The mean pre-induction temperature of all the 300 patients was 36.54°C. The regression analysis line fit plot between pre-induction temperature and intra-operative temperature predicts that if the mean pre-induction temperature is raised to 37.2°C, the predicted intra-operative temperature will be 36.6°C.

**Figure 5:**
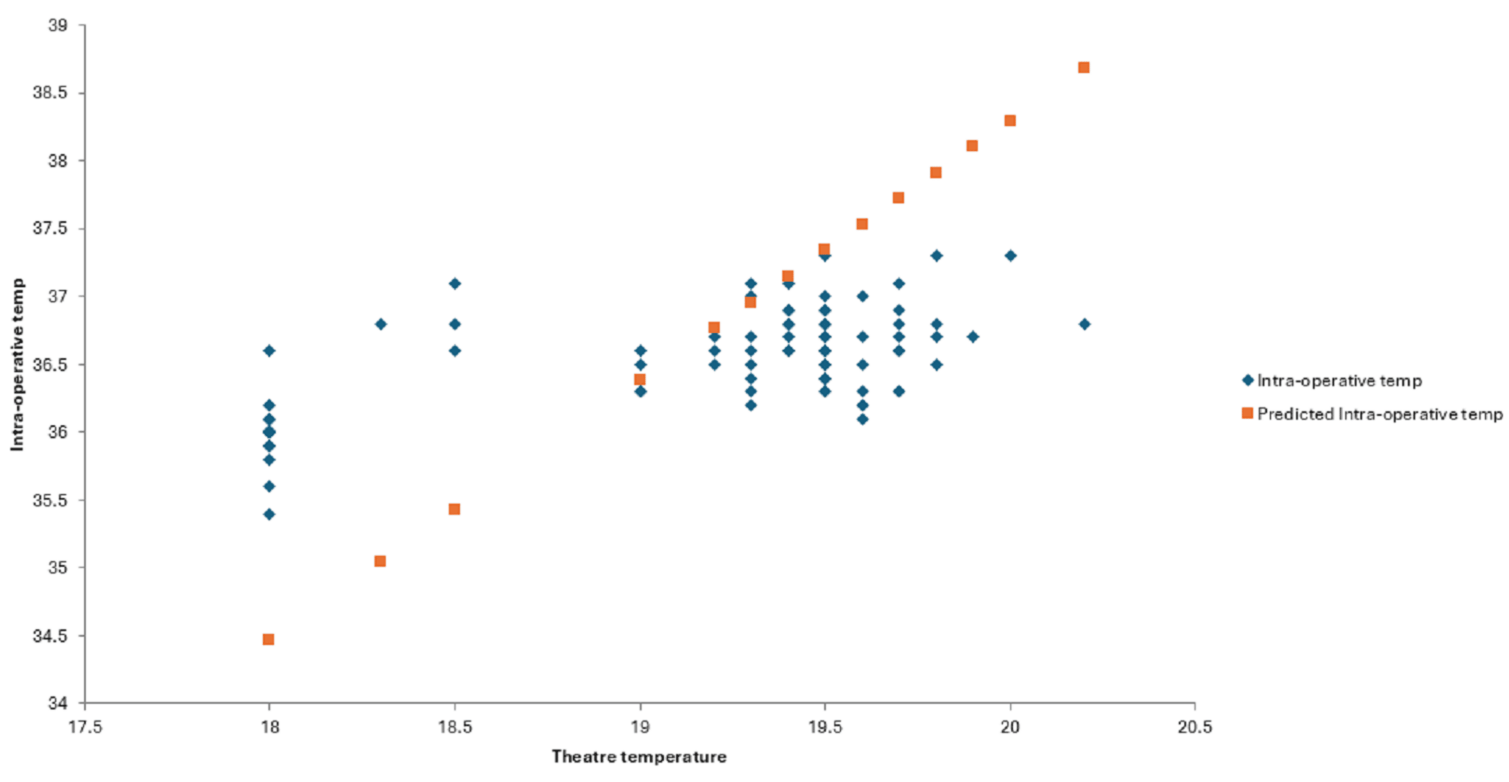
Line fit plot between the theatre ambient temperature (in °C) and intra-operative temperatures (in °C)

Multivariate regression analysis of intra-operative and theatre ambient temperatures resulted in a p value of <0.001 (Figure 5). This suggests that the theatre ambient temperature is statistically significant parameter for predicting intra-operative hypothermia. The mean operating room ambient temperature for all 300 cases was 19.4°C. The regression analysis line fit plot between theatre ambient temperature and intra-operative temperature predicts that if the mean theatre temperature is raised to 19.9°C, the predicted intra-operative temperature will be 37°C. The line fit plot also predicts that if the ambient operating room temperature is 18°C, the predicted intra-operative temperature will be 34.4°C. If the operating room temperature is 19°C, the predicted intra-operative temperature will be 36.3°C, and at 20.2°C, it will be 37.6°C.

## Discussion

According to a recent study, the average cost of each trauma case in NHS is about £7,553, mostly consisting of ward and staffing costs. The calculated mean ward cost per patient per day was £143.20 on an orthopaedic ward, £131.93 on an inpatient rehabilitation ward and £559.42 on a High Dependency Unit/Critical Care Unit setting [33]. It is, hence, imperative to identify the modifiable factors for the prevention of complications and reduction of inpatient hospital stays. It is well known that perioperative hypothermia increases the risk of infection and duration of hospital stay in multiple groups, including colorectal surgery patients, trauma patients and orthopaedic patients [34-38].

Inadvertent intra-operative hypothermia is the most common thermal disturbance perioperatively. The incidence of this varies with the surgical population and patients' demographics. Old age, severe burns, severe trauma, low pre-operative temperature and a major shift of fluid intra-operatively are pre-disposing factors leading to a higher risk of hypothermia [39-41]. Studies have shown that the incidence of perioperative hypothermia is higher in female patients [40], and though our study had more female patients who developed intra-operative hypothermia, sex was not a statistically significant factor in the development of intra-operative hypothermia.

The benefits of maintaining normothermia, that is, core body temperature of 36°C-38°C throughout the peri-operative phase, have been well delineated [35]. Perioperative normothermia is recommended by the World Health Organisation, National Quality Forum, Surgical Care Improvement Project and National Institute for Health and Care Excellence 65 guidelines [32,42-45]. Complying with the peri-operative normothermia standards does not always result in achieving normothermia. A recent retrospective study, which included over 10,000 surgical patients, concluded a 5.8% rate of post-operative hypothermia even though hypothermia prevention methods were employed in 97.9% of the cases. The cohort of over 4,000 orthopaedic patients included in the study experienced an increased rate (7.7%) of hypothermia upon admission to the post-anaesthesia care unit despite a 99.3% compliance rate to hypothermia prevention methods [44]. Thus, it may be difficult to achieve peri-operative normothermia in orthopaedic patients.

The primary goal of our study was to examine the incidence of hypothermia in trauma patients in various phases of surgery and, most importantly, the intra-operative phase, as it has been strongly linked to post-operative complications, including surgical wound infection, sepsis, cardiovascular complications and longer hospital stay [3-7] adding to the financial burden on the NHS. Our incidence of post-operative hypothermia of 21% was lesser than that published by Leijtens et al., who reported a 27% hypothermia rate in total knee and hip arthroplasty [45]. Our incidence of intra-operative hypothermia of 21% was lesser when compared to the 32% reported by Matos et al., though these were reported in patients who were undergoing elective joint arthroplasty [46]. This can be attributed to the fact that about 71% of our patients had regional anaesthesia, which can lead to a decreased incidence of intra-operative hypothermia when compared to general anaesthesia [47]. A perceived bias may exist in patients receiving regional anaesthesia, who would be more likely to complain of feeling cold. This leads the anaesthesia team to implement warming strategies early in such patients, thereby reducing the incidence of intra-operative hypothermia. Our current practice with patients undergoing regional as well as general anaesthesia is to implement active warming of the patients using forced air warmers, and temperature measurement is done by using tympanic thermometers in all patients.

Our study showed that patients who undergo upper and lower trauma surgeries under general anaesthesia have a higher incidence of hypothermia when compared to regional anaesthesia. These findings are in line with the recent large retrospective study on patients undergoing lower extremity joint arthroplasty that reported a higher rate of hypothermia with general anaesthesia when compared to regional anaesthesia [48]. Though general anaesthesia is typically associated with increased rates of peri-operative hypothermia, neuraxial anaesthesia typically results in impaired thermoregulation in both central and autonomic nervous systems [49].

The pre-induction temperature, as well as the theatre ambient temperature, has been by far the most statistically significant factor in the development of intra-operative hypothermia. A recent study by Habib et al. suggested that if the pre-induction temperature of elective surgical patients is at a thermal comfort zone of 37.2°C, the predicted intra-operative temperature would be 36.74°C [41]. The multivariate regression analysis of our study projects that if the pre-induction temperature of patients undergoing trauma surgery is raised to 37.2°C, the predicted intra-operative temperature is 36.6°C. Several studies have shown that pre-induction warming results in minimising intra-operative hypothermia [26,41,50-52]. Operating theatre ambient temperature plays a major role in the development of intra-operative hypothermia as cooler surroundings lead to a greater radiation loss of heat from the exposed tissue surfaces during surgeries [53,54]. Our study highlights the importance of tight regulation of theatre temperature to prevent intra-operative hypothermia. A trial of pre-warming of the operating theatres before induction of anaesthesia in paediatric patients had concluded that there was a benefit of pre-warming due to reduced decline in the core body temperature of the patients in the treated group, and the benefit was greatest for cases lasting less than 45 minutes [30].

An unexpected finding of the study was that BMI was not a statistically significant factor in the development of intra-operative hypothermia. Even though it was postulated that a higher BMI would protect patients from heat loss, this association was not found. In contrast to our study, many studies have found a positive correlation between patient BMI and intra-operative temperature difference and a smaller decrease in core body temperature in patients with higher body fat content [55,56].

The study had several limitations, including a small number of patients, and no power calculation was done to determine the sample size. The study was designed to study the incidence of perioperative hypothermia in trauma patients and to determine if intervention was warranted. The post-operative complications, length of stay, readmissions and duration of the hypothermia episode were not assessed. One observational study involving 120 patients undergoing elective knee or hip arthroplasty reported that over 20% of the patients were hypothermic for longer than one hour [46]. The duration of hypothermia and associated effects on the length of hospital stay and post-operative complications need to be studied in greater detail in upcoming studies.

The ambient room temperatures of the pre-operative and recovery rooms were not recorded, and this may have influenced the patients' pre-induction and post-recovery temperatures. The intra-operative use of different warming devices was not considered and will need to be further investigated in the future studies.

## Conclusions

This study highlights the need for active interventions to recognise and prevent perioperative hypothermia in patients undergoing trauma surgeries. The incidence of hypothermia is high in trauma patients, and more can be done to prevent this. Pre-warming of trauma patients can be undertaken to reduce the risk of intra-operative hypothermia, as the pre-induction temperature is a statistically significant factor in our study. The study also highlights the importance of ambient operating room temperature, which is shown to be statistically significant. Further studies can include a randomised controlled trial comparing active and passive warming strategies and pre-warming of the operating rooms in trauma patients. The goal is to determine if active or passive patient warming and pre-warming of operating rooms is more effective in trauma patients. Such a trial will assess outcomes, including core body temperature, thermal comfort, length of overall hospital stay and complication rates.
